# Monitoring and Characteristics of Mpox Contacts, Virginia, USA, May–November 2022

**DOI:** 10.3201/eid3003.230609

**Published:** 2024-03

**Authors:** Eleanor N. Field, Elizabeth McCarty, Dawn Saady, Brandy Darby

**Affiliations:** Centers for Disease Control and Prevention, Atlanta, Georgia, USA (E.N. Field);; Virginia Department of Health, Richmond, Virginia, USA (E.N. Field, E. McCarty, D. Saady, B. Darby)

**Keywords:** monkeypox virus, mpox, response, outbreak, contact tracing, viruses, Virginia, United States

## Abstract

During 2022, a global outbreak of mpox resulted primarily from human-to-human contact. The Virginia Department of Health (Richmond, VA, USA) implemented a contact tracing and symptom monitoring system for residents exposed to monkeypox virus, assessed their risk for infection, and offered interventions as needed. Among 991 contacts identified during May 1–November 1, 2022, import records were complete for 943 (95.2%), but 99 (10.0%) were not available for follow-up during symptom monitoring. Mpox developed in 28 (2.8%) persons; none were healthcare workers exposed at work (n = 275). Exposure risk category and likelihood of developing mpox were strongly associated. A total of 333 persons received >1 dose of JYENNOS (Bavarian Nordic, https://www.bavarian-nordic.com) vaccine, most (n = 295) administered after virus exposure. Median time from exposure to vaccination was 8 days. Those data tools provided crucial real-time information for public health responses and can be used as a framework for other emerging diseases.

Mpox is an emerging viral disease characterized by a prodromal illness followed by vesiculopustular rash (*1*). Since monkeypox virus (MPXV) was first isolated in 1970 from a child in the Democratic Republic of the Congo, cases of mpox have been documented across 15 countries, primarily Africa (*1*). Sporadic cases outside of those countries were usually epidemiologically linked to international travel or animal importation (*2*). However, during 2022, a global outbreak of mpox began that was driven by human-to-human transmission (*3*,*4*); ≈87,000 cases from 110 countries have been reported to the World Health Organization since January 2022 through May 2023 (*5*). Before 2022, no mpox case had been reported in Virginia, USA; however, by the end of December 2022, Virginia reported 568 cases and was among the top 15 US states for mpox case burden (*6*).

In Virginia, mpox is reportable as an Unusual Occurrence of Disease of Public Health Concern. Local public health departments have 24 hours from case notification to begin an investigation, initiate contact tracing to identify exposed persons, and offer medical countermeasures to halt further transmission. The 2-dose vaccine series (JYNNEOS; Bavarian Nordic, https://www.bavarian-nordic.com) was offered for persons at increased risk for MPXV exposure or after a known or presumed exposure to MPXV (*7*). The Centers for Disease Control and Prevention (CDC) recommends that vaccine be given as soon as possible, ideally within 4 days after exposure; administration 4–14 days after exposure may still provide some protection against mpox and should still be offered (*7*). The second dose should be administered 28–35 days after the first dose, although completing the series at any time thereafter is recommended (*7*).

The changing epidemiology of MPXV transmission from primarily zoonotic to primarily human-to-human during an outbreak of unprecedented scale provided a unique public health challenge. We describe how the Virginia Department of Health (VDH; Richmond, VA, USA) adapted an existing data collection tool for tracing contacts and monitoring symptoms of persons affected by an emerging disease and how those data were used to assess contact characteristics, MPXV exposures, vaccine uptake, and timeliness of postexposure vaccination.

Our study received ethics approval from the Virginia Department of Health Institutional Review Board (study #50284). The study was also reviewed by CDC and conducted consistent with federal law and CDC policy (*45 C.F.R. part 46, 21 C.F.R. part 56; 42 U.S.C. Sect. 241(d); 5 U.S.C. Sect. 552a; 44 U.S.C. Sect. 3501 et seq.).

## Materials and Methods

### Cohort Design

The objective of VDH mpox contact tracing was to identify close contacts, advise them of the virus exposure, and offer vaccination to prevent illness or reduce disease severity to those eligible. Symptom monitoring was implemented to expedite early laboratory testing and case identification to reduce further transmission. To be included in the study, a person needed to have either self-reported an MPXV exposure or have been notified by VDH of a recent exposure. Persons who were not residents of Virginia were not eligible for participation. VDH may have been notified of an mpox case by an in-state healthcare provider, clinic, or laboratory; by another state; or by CDC.

We recorded persons with confirmed and probable mpox identified during the symptom monitoring period as persons in whom mpox developed. We defined a confirmed mpox case as positive detection of MPXV through either molecular testing or genomic sequencing. We defined a probable case as detection of orthopoxvirus by molecular testing and no laboratory evidence of another nonvariola orthopoxvirus, detection of orthopoxvirus by immunohistochemistry or genomic sequencing, or detection of orthopoxvirus IgM in a person with no recent history of vaccination (*8*).

### Mpox Contact Tracing and Symptom Monitoring Data Collection

Local health department staff used REDCap (Research Electronic Data Capture, https://www.project-redcap.org) to collect information on mpox close contacts and symptom monitoring during case and contact interviews. Some hospitals monitored their own employees and provided information to local health departments about their healthcare workers (HCWs) exposed at work. Information was entered into a contact import form that included patient demographics, MPXV exposure (e.g., date of last exposure, exposure risk category, location description and setting), mpox vaccination status, HCW status, immunosuppression status, and public health interviewer details. We also linked close contact to a daily mpox monitoring form, which collected information about mpox symptoms (e.g., temperature, rash, chills, swollen lymph nodes), medications taken, and final disposition. The daily mpox monitoring form was completed and submitted by the contact over text message, email, or by phone with a local health department staff member.

The REDCap project also included a case report form, which was adapted from CDC recommendations (*9*). The form consisted of 248 fields asking about the interaction(s) that may have been the source(s) of infection, mpox vaccination status, mpox hospitalization, mpox symptoms, date of illness onset, residence, demographics (including sexual orientation and gender identity), recent trips and contacts with whom the person had interacted (and the nature of the interactions), laboratory information about the diagnosis, and interview details.

Contact information obtained from case interviews was recorded in the database, but participation in daily mpox symptom monitoring and exposure or case interviews with the local health department was voluntary. Symptom monitoring lasted for 21 days from a person’s last reported exposure.

### Cohort Analyses

**Figure 1:**
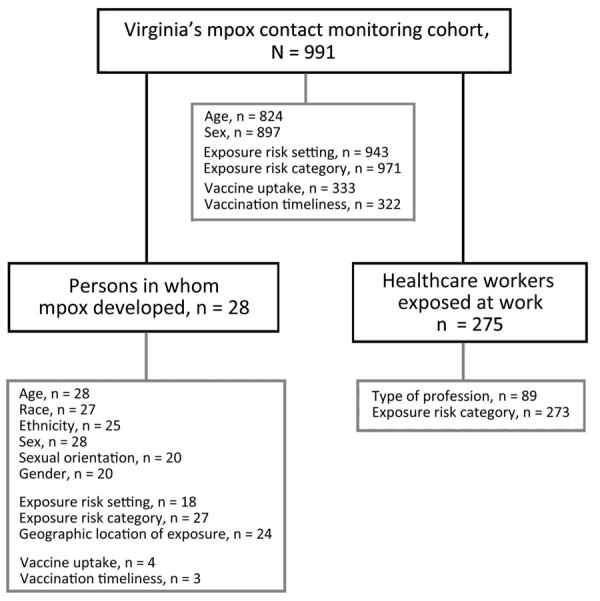
Mpox contact tracing and symptom monitoring cohort (n = 991), Virginia, USA, May 1–November 1, 2022. Analyzed subcohorts included persons in whom mpox developed (n = 28) and healthcare workers exposed at work (n = 275).

We conducted a retrospective cohort study for persons enrolled in the VDH mpox close contact monitoring cohort during May 1–November 1, 2022 (Figure 1). We excluded data for 16 persons who had not completed symptom monitoring within the study time frame and for 1 person for whom duplicate, conflicting information was recorded. For all analyses, we used R Statistical Software version 4.2.2 (The R Foundation for Statistical Computing, https://www.r-project.org).

### Mpox Exposure Analysis

We extracted information regarding demographics, MPXV exposure details, assigned exposure risk category (*10*), monitoring participation, and outcome (disease did vs. did not develop) of persons included in the monitoring cohort. Exposure settings were mutually exclusive because of limitations in the structure of data collection forms. Exposure risk categories (high, intermediate, lower, and none) characterizing personal risk from the nature of the exposure using criteria defined by CDC (*10*) were assigned by local health department personnel in the contact import form.

We used descriptive statistics to describe select demographic and exposure data for the full cohort, for persons within the cohort in whom mpox developed, and for HCWs exposed at work (Figure 1). Calculated percentages exclude missing values. We used χ^2^ square analysis to evaluate the association between exposure risk category (excluding the none category) and development of mpox.

### Mpox Vaccination Analysis

Mpox vaccine administration is mandatorily reported to the Virginia Immunization Information System (https://viis.vdh.virginia.gov); we used this system to determine which persons received in-state mpox vaccine(s) and the date(s) of administration. Matching was completed by using exact date of birth, postal (ZIP) code, and the first 3 letters of first and last names.

To assess vaccine uptake, we described how many and what percentage of persons within the cohort received >1 dose of an mpox vaccine. We used those descriptive statistics to measure completion of the 2-dose series. We also specifically assessed vaccine uptake for persons within the cohort in whom mpox developed. Last, to determine if there were differences across exposure risk categories, we measured vaccine uptake by exposure risk category.

We measured vaccination timeliness as time in days from reported MPXV exposure to first dose of an mpox vaccine for the full cohort and for persons in whom mpox developed. We did not analyze preexposure vaccination timeliness. We also assessed timeliness by using CDC postexposure recommendations (*7*), describing how many doses were administered within 4 and 14 days of the reported exposure.

## Results

### Cohort Characteristics

During May 1–November 1, 2022, a total of 991 persons were enrolled in Virginia’s mpox close contact monitoring cohort and ended their 21-day monitoring period during the study period. Among the 932 persons for whom data about their method of participation were available, 491 (52.7%) used email, 239 (25.6%) reported directly to their local health department, 143 (15.3%) self-monitored, and 59 (0.06%) used text messaging to access surveys. Of 991 contact records, 943 (95.2%) were complete and 48 (4.8%) were incomplete. During symptom monitoring, 99 (10.0%) contacts were not available for follow-up and 20 (2.2%) declined or no longer needed monitoring (e.g., their reported exposure was beyond the 21-day symptom monitoring period, not determined to be a close contact, or from a person later determined to be MPXV negative). Eleven (1.1%) contact investigations were transferred to another jurisdiction. Of the 28 persons in the close contact monitoring cohort in whom mpox developed, 26 (92.9%) completed their case interview. Among 897 persons in the cohort for whom sex was recorded, 494 (55.1%) were male and 403 (44.9%) female (Table 1). Age information was available for 824 persons; median age was 35 (interquartile range [IQR] 26–49) years.

**Table 1:** Characteristics of 991 persons enrolled in mpox contact tracing and symptom monitoring cohort, Virginia, USA, May 1–November 1, 2022*

		No. (%) persons		
Characteristic		Total	Persons without mpox	Persons with mpox
Total		991	963	28
Sex assigned at birth				
M		494 (55.1)	467 (53.7)	27 (96.4)
F		403 (44.9)	402 (46.3)	1 (3.6)
Missing		94	94	0
Age group, y				
0–9	32 (3.9)		32 (4.0)	0 (0)
10–19	48 (5.8)		48 (6.0)	0
20–29	130 (15.8)		128 (16.1)	2 (7.1)
30–39	205 (24.9)		193 (24.2)	12 (42.9)
40–49	155 (18.8)		145 (18.2)	10 (35.7)
50–59	101 (12.3)		99 (12.4)	2 (7.1)
60–69	103 (12.5)		101 (12.7)	2 (7.1)
70–79	0 (4.4)		36 (4.5)	0
>80	14 (1.7)		14 (1.8)	0
Missing	167		167	0

*Calculated percentages exclude missing values.

### Persons with Mpox Cohort Characteristics

Within the cohort of 991 persons, mpox developed in 28 (2.8%) while they were being monitored for symptoms (Figure 1); 27 cases were confirmed and 1 was probable. Twenty-seven (96.4%) persons were recorded as male and 1 (3.6%) as female (Table 1). The median age was 36 (IQR 31–40) years. Among 27 persons with mpox who reported their race, 15 (55.6%) self-identified as White, 11 (40.7%) as Black, and 1 (3.7%) as Native Hawaiian or Other Pacific Islander. Among 25 persons with mpox who reported ethnicity, 8 (32.0%) self-identified as Hispanic. Information on sexual orientation and gender identity was available for 20 persons with mpox; 19 (94.7%) self-identified as bisexual or gay cisgender men, and 1 (5%) self-identified as a straight cisgender woman.

### Reported Mpox Exposure Settings

**Figure 2:**
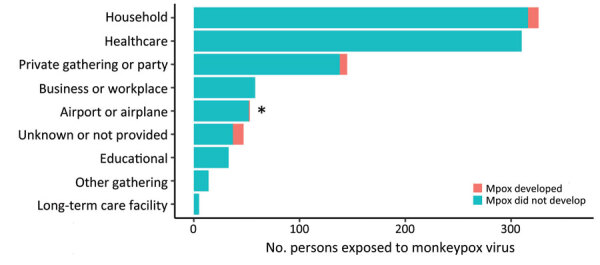
Reported monkeypox virus exposure setting categories from mpox contact tracing and symptom monitoring cohort (n = 991), Virginia, USA, May 1–November 1, 2022. For persons in whom mpox developed while being monitored (n = 28), asterisk indicates where initial reported exposure setting differed from most likely infection source.

Exposure information was available for 943 persons in the cohort (Figure 1). Of those, 326 (34.5%) were exposed in households, 310 (32.9%) in healthcare settings, 145 (15.4%) at private gatherings or parties, 58 (6.2%) in workplaces, 52 (5.5%) in an airport or airplane, 33 (3.5%) in a school, 14 (1.5%) in other congregate settings, and 5 (0.5%) in a long-term-care facility (Figure 2).

### Reported Mpox Exposures in Persons in Whom Mpox Developed

Reported exposure setting information was available for 18 of the 28 persons in whom mpox developed; 10 reported MPXV exposures from a household (55.6%) and 7 from a private gathering or party (38.9%). One (5.6%) person was being monitored for exposure on an airplane or in an airport, but investigators later determined that that was not the most likely source of infection (Figure 2).

Among the 25 persons in whom mpox developed and who provided additional information about their MPXV exposure, 22 (88.0%) reported recent sexual activity. Seven men reported sexual activity with multiple male partners, and 3 of them reported that their partners were anonymous. Mpox developed in 1 straight cisgender woman within the cohort after a reported sexual exposure from a male household contact. Of the 3 persons in whom mpox developed without their having reported recent sexual contact, 2 persons reported that their exposure was from a congregate setting (specifically, a prison and a convention event) and 1 person reported close nonsexual contact. Geographic exposure location was available for 24 persons: 18 (75.0%) reported in-state exposures and 6 (25.0%) reported exposures during out-of-state domestic travel (to Georgia, North Carolina, New York, and Massachusetts) or international travel (Mexico).

### Mpox Exposure Risk Categories

Among 971 persons for whom exposure risk categories were assigned by using CDC criteria (*10*) (Figure 1), 374 (38.5%) were assigned intermediate risk, 360 (37.1%) lower risk, 225 (23.2%) higher risk, and 12 (1.2%) no risk (Table 2). Among the 28 persons in whom mpox developed for whom an exposure risk category was assigned, 20 (71.4%) exposures were categorized as high risk, 4 (14.3%) as intermediate risk, and 3 (10.7%) as lower risk; 1 person (3.6%) was not assigned an exposure risk category (Table 2). The degree of association between assigned exposure risk category and likelihood of mpox development was high (p<0.001) (Table 2).

**Table 2:** Exposure risk categories and likelihood of developing mpox among 991 persons included in mpox contact tracing and symptom monitoring cohort, Virginia, USA, May 1–November 1, 2022*

Risk category	No. (%) persons			χ^2^ (d.f.)†	p value
	Total	Persons without mpox	Persons with mpox		
None	12 (1.2)	12 (1.3)	0		
Lower	360 (37.1)	357 (37.8)	3 (11.1)	39.7 (2)	<0.001
Intermediate	374 (38.5)	370 (39.2)	4 (14.8)		
High	225 (23.2)	205 (21.7)	20 (74.1)		
Missing	20	19	1		

*Calculated percentages exclude missing values. Predetermined categories were defined by the nature of the exposure using criteria defined by the Centers for Disease Control and Prevention (*10*). †χ^2^ analysis excludes persons in the none category.

### HCW Occupational Exposures

A total of 275 persons self-identified as HCWs who were exposed at work (Figure 1). Among the HCWs who reported their role, 2 (2.1%) were administrators, 14 (15.4%) worked in emergency medical services, 1 (1.1%) was an imaging technician, 27 (29.7%) were nurses, 7 (7.7%) were nurse assistants, 14 (15.4%) worked as other direct care HCWs, 1 (1.1%) worked as an other nondirect care HCW, 21 (23.1%) were healthcare providers, and 3 (3.3%) worked in registration. Among 273 HCWs exposed at work for whom an exposure risk category was assigned, 34 (12.5%) exposures were categorized as high risk, 48 (17.6%) as intermediate risk, 180 (65.9%) as low risk, and 11 (4.0%) as no risk (e.g., personal protective equipment was appropriately worn during exposure encounter[s]).

### Vaccine Uptake

Of the 991 persons in the cohort, 333 (33.6%) received >1 vaccine dose that was recorded in Virginia’s Immunization Information System (Table 3; Figure 1). In addition, 212 received a second dose, representing 63.7% of those available for follow-up and indicating that 21.4% of the cohort completed the mpox series during May–November 2022.

**Table 3:** Vaccine uptake and postexposure timeliness in mpox contact tracing and symptom monitoring cohort, Virginia, USA, May 1–November 1, 2022*

Characteristic	Value
All persons	991
Received >1 dose	333 (33.6)
Before exposure	27 (8.4)
After exposure	295 (91.6)
Unable to determine	10
Received 2 doses	212 (63.7)
Persons vaccinated after exposure	295
Median time from exposure to first dose, d	8 (range 4**–**12)
No. receiving 1st dose within **<**4 days of exposure	82 (27.8)
No. receiving 1st dose within **<**14 days of exposure	252 (85.4)

*Values are no (%) except as indicated. Calculated percentages exclude missing values.

Of the 225 persons identified as having had a high-risk exposure, 121 (53.8%) received >1 dose. A total of 166 (44.3%) of 374 persons who had intermediate risk exposures received >1 dose, and 35 (9.7%) of 360 persons self-identified as having lower exposure risk received >1 dose.

Information about exposure and vaccination dates were available for 322 of the 333 vaccinated persons. A total of 295 (91.6%) persons received postexposure vaccination, and 27 (8.4%) received preexposure prophylaxis (Table 3).

### Timeliness of Postexposure Vaccination

Among the 295 persons who received postexposure vaccination, the median time of first vaccine administration after MPXV exposure was 8 (IQR 4–12) days (Table 3). In terms of timeliness of recommended postexposure administration, 82 (27.8%) persons were vaccinated <4 days after MPXV exposure and 252 (85.4%) were vaccinated <14 days after exposure (Table 3). Information on exposure and vaccination dates were available for 3 of the vaccinated persons in whom mpox developed; all had received postexposure prophylaxis within 14 days (4, 11, and 12 days).

## Discussion

The data tool that we used enabled flexibility and for real-time review of data from personnel at the local and state health department level to track the number of persons who had been exposed to MPXV and offer interventions to persons at high risk for exposure to stop transmission. Contact lists were easily exported so that health department personnel could cross-check against Virginia’s vaccine registry to encourage vaccination completion. The overall high completion rate of contact records and low number of persons not available for follow-up during symptom monitoring demonstrates successful implementation and use of the VDH mpox close contact monitoring response.

We found no cases of mpox in HCWs exposed at work. Most exposures for HCWs were lower risk, potentially suggesting either some use of personal protective equipment or minimal contact with the patient. Details about high-risk exposures in medical settings were not provided and could be an area of further research. Similarly, mpox did not develop in any persons exposed in businesses, workplaces, or educational settings. We do report mpox development after household exposures, but case interviews more specifically identified that the source of infection was from sexual contact in a household environment rather than cohabitation with an infected person. That finding is consistent with results from a recent study of undiagnosed mpox prevalence in the United States (*11*).

The high degree of association between assigned exposure risk category and likelihood of mpox development suggests that risk categories are useful for public health officials identifying persons to prioritize for interventions. Our cohort analysis identified 3 persons who were labeled lower risk but in whom mpox developed. One person disclosed sexual contact unrelated to known exposure, and it is likely that the assigned classification instead reflected the exposure for which the person was being monitored. One person disclosed recent sexual contact without other potential exposure sources and represents a misclassification of exposure risk category, underrepresenting mpox risk. The third person did not complete an interview, so it is unclear how that risk category was assigned.

Overall vaccine uptake in this cohort was low; only one third of the cohort received >1 dose and one fifth completed the 2-dose series. Just over half of persons who were identified as having had a high-risk exposure received a vaccine. However, more persons categorized as having high-risk exposure were vaccinated than were persons in other exposure risk categories, which might suggest higher motivation to receive vaccination or success in vaccine prioritization.

Timely vaccine uptake for postexposure prophylaxis was low; <30% of persons were vaccinated within the recommended 4 days after a known or presumed MPXV exposure. However, most (85%) persons who received postexposure vaccine received it within 14 days of their exposure, which may confer some protection (*6*). Factors such as reduced patient access to diagnostic testing may have delayed the initial mpox case-patient’s diagnosis, affecting exposure notification to contacts. In addition, vaccine availability might have affected vaccination timeliness.

Among the limitations of our retrospective cohort analysis, persons exposed to MPXV or who had mpox might have been missed by official VDH reporting channels, and we were unable to estimate how well our cohort captured these populations. Also, persons with mpox interviewed by public health personnel may have been hesitant to discuss sexual exposure details, leading to underreporting and lack of follow-up with contacts or misclassification of infection risk.

In conclusion, our study describes mpox contact tracing and symptom monitoring in Virginia and evaluated characteristics of persons with reported exposures and can be used to inform public health preparedness and response measures. The flexible data collection tools and real-time access to data used by VDH in the mpox response can serve as a framework for future emerging diseases.
